# Nonhuman Mobilities and Multispecies Border Regimes in (Post‐)Colonial Southern Africa**

**DOI:** 10.1002/bewi.70033

**Published:** 2026-08-23

**Authors:** Luregn Lenggenhager

**Affiliations:** ^1^ Global South Studies Centre (GSSC) University of Cologne Cologne Germany; ^2^ Centre for African Studies University of Basel Basel Switzerland

**Keywords:** Borders, Colonialism, Livestock, Multispecies Histories, Nature Conservation, Southern Africa

## Abstract

This article explores how borders in Southern Africa—such as Namibia's internal veterinary and settler border (Red Line) and national borders between Namibia, South Africa, and Botswana—functioned not only as territorial dividers but as tools to shape colonial and postcolonial multispecies hierarchies. These borders regulated human and animal movement, reinforcing racial and economic inequalities by privileging certain species and people while restricting or dehumanizing others. This study examines political, veterinary, and conservation borders to show how they naturalized racist multispecies hierarchies and legitimized colonial oppression. In doing so, it reveals the dangers of blurring the boundaries between human and nonhuman life, as well as the importance of recognizing nonhumans as co‐constituting borders and boundaries. I argue that more‐than‐human historiography must critically engage with the local histories of border regimes and animal movements to understand their ongoing role in reproducing multispecies power imbalances in (post‐)colonial contexts.

## 1 Introduction

“Nature knows no boundaries” is one of the most overused and oversimplified buzz slogans in conservation circles worldwide. From WWF's transalpine conservation program in Europe, to the large corporation‐led partnership Oceans Without Borders (OWB), to the mushrooming transfrontier conservation areas in Southern Africa, to smaller organizations such as Elephants Without Borders (EWB) in Botswana—they all use these and similar slogans to promote their conservation initiatives. It does not need much ecological, political or historical analysis to question the validity of such slogans. Of course, nature *knows* boundaries, be it the boundaries of species distribution, boundaries of landscape and land‐use features or any other boundaries along which natural features are organized. However, as obvious as this is, it is also unquestionable that physical and political borders have always been interacting closely with nature, restricting and allowing movements of human and nonhuman life as well as the introduction or removal of certain species, and also in creating, upholding and challenging boundaries between and within different species and groups of species. This article shows that these inter‐ and intraspecies boundaries have been closely linked to political borders and the establishment of multispecies hierarchies, particularly in colonial and apartheid Southern Africa. I argue that political boundaries were crucial in the establishment of hierarchies within and between groups of humans and nonhumans. Today, these historically shaped boundaries are reflected in shifting patterns of human and nonhuman presence and interactions, where the dominance of certain species is increasingly experienced as a loss of others—altering not only entire systems of interactions between and within humans, animals and plants, but also local perceptions of multispecies coexistence.

I start with a short conceptual engagement with the role and function of borders in more‐than‐human or multispecies history writing in the (post‐)colony and show that applying a multispecies approach in a society that has been based on the de‐humanization of certain (Black) people and the celebration of certain (charismatic) species needs to be done with particular care for localized, specific contexts. I will then introduce three interlinked case studies from Southern Africa: the veterinary and settlement border of Namibia's Red Line through the mobility of donkeys, the border regimes surrounding Karakul sheep in southern Namibia, and the conservation and national border along the Chobe River between Namibia and Botswana. Together, these cases show how borders not only hierarchize humans, animals, and plants through shifting regimes of valuation, but are themselves shaped, contested, and reconfigured through multispecies mobilities and everyday practices.

## 2 More‐than‐Human Histories, Border Studies and Colonialism

Over the past decades, climate change, the continuing commodification of nature and new biological conceptualizations of nonhuman life have moved to the forefront of public and scholarly concern.^1^ Concurrently, the humanities in general and history in particular had to rethink their understanding of the role of humans within the web of life and to put more focus on the role of nonhuman actors.^2^ However, the concept of a more‐than‐human or multispecies society has a long and violent history in the (post‐)colony, which needs to be carefully weighed‐in to critical animal history, to avoid reinforcing colonial and apartheid concepts of human‐nonhuman relations. A questioning of the human‐animal divide, as initially suggested by scholars such as Richard White, argues that environments, nature, and landscape are merely culturally constructed, that their material importance for history and society must be relativized, and the lines between culture and nature should be blurred.^3^ This perspective was not only criticized for being depoliticizing and neglecting biological and economic contexts but also for reinforcing a Western hegemony.^4^ Critics argue that it overlooks the colonial history of the muddying of the divide between culture and nature.^5^ This, of course, is a highly condensed and limited account of a central strand of debate around the construction of Western modernity—one in which Africa has been variously framed as its constitutive outside, its material foundation, its invention, or a site of active engagement with alternative, entangled, or vernacular forms of modernity.^6^ Such foundational questions in debates on Western modernity have also become increasingly debated through multispecies research that interrogates the agency of nonhuman actors and its role in colonial and imperial scientific contexts.^7^ As I will show, an engagement with these debates crucially benefits from an empirically nuanced, time‐ and space‐sensitive analysis of specific human‐nonhuman interactions.^8^

Generally, a questioning of the human‐animal divide was received with scepticism in African academic contexts, because it was seen as worryingly close to racist, colonial and apartheid conceptualizations of African people either as mere ‘products of nature’ or as a danger to an imagined pristine nature.^9^ Such histories of dehumanizing Black people still engender (Southern) African understandings of animal‐human relations up to the present day.^10^ Frantz Fanon highlighted that “[i]n plain talk, he [the colonized] is reduced to the state of an animal.”^11^ This ideology is fundamentally rooted in a worldview that hierarchizes both human and nonhuman life. Rather than positioning all humans above all animals, it establishes intersecting hierarchies in which (white, male) humans assume the role of protectors and saviours of a “debased” nature.^12^ Within this framework, hierarchies between animals—ranging from *vermin* to *royal game*—are so deeply intertwined with the racial categorization of humans that even the category “human” itself becomes contested.^13^ Such a worldview made forced removals or even the “eradication” of certain categories of humans look “natural.”^14^

In the same vein, the intensely debated questions around nonhuman agency also have another layer of concern when viewed in a context where powerful humans have actively denied agency—or, to use Amanda Rees’ words, “the capacity to contribute to the future”—to certain humans (and certain animals), while hardly questioning that some key animals, such as commercial livestock or charismatic wildlife, possess “the ability to change the outcome of events.”^15^

Neglecting these racist and imperial contexts in more‐than‐human debates shows, according to scholars such as Maneesha Deckha, that current debates about human‐animal relations have long been trapped within “colonial logics fortifying a reified Western/non‐Western binary and civilizational discourses positioning Western culture as superior to non‐Western cultures.”^16^ Addressing such concerns also calls for greater attention to material and political border zones, where more‐than‐human entanglements both shape and are shaped by spatial regimes of control, demarcation, and exclusion.

In the wide field of border and borderland studies, at least from the perspectives of geographers and social and economic historians, the role of nonhuman life only recently gained more interest, intensified by the spread of the COVID‐19 pandemic revealing the global entanglements between animals, viruses and border control.^17^ Understanding geographical and political borders as contact zones highlights bordering as co‐constituted by humans and nonhuman actors like animals, viruses, plants, and technologies. The term “contact zone,” coined by Mary Louise Pratt, originally described social spaces where cultures meet, clash, and negotiate amid uneven power during imperial expansion. Donna Haraway later expanded this to “multispecies contact zones,” including nonhumans to challenge narrow views of relatedness and power.^18^

A more‐than‐human approach to border studies opens up new lines of inquiry for the study of borders and connectivity beyond an anthropocentric scope, while still offering a focal point of re‐thinking material and political hierarchies within and between species.^19^ While animals, or nonhumans more broadly, play roles in both stabilizing and destabilizing borders, these processes also reciprocally stabilize or destabilize various categories encompassing both humans and nonhumans.^20^ This dynamic has sparked intense debate across disciplines, from STS, to critical animal geographies, and also in the fields of biology and history of biology.^21^ However, most studies concern themselves with the current situation, contributing to general, global conceptual approaches often focusing on endangered species and relations of care.^22^ Thoroughly researched empirical small‐scale analyses of historical cases are often missing. Only recently, research on the “continuous significance of nonhuman bodies to the operation of power” along political and geographical borders—or contact zones—emerged.^23^ Such social, economic, and political histories of contact zones along borders show both the active role of nonhuman actors in destabilizing border regimes and the active use of nonhumans to control, survey, and uphold them.^24^

In the following, I will show how geographical and political borders have been continuously transgressed by both humans and nonhumans and how these crossings altered or perpetuated validations of certain species and groups of humans. This happened on various levels during colonial and apartheid times in Southern Africa, and impacts transboundary human‐nonhuman interaction until today. An engagement with animals—or generally nonhuman beings—as both constituting and traversing borders can also be analysed in dialogue with the concept of boundary objects developed by Star and Griesemer.^25^ After all, the animals and plants I describe enabled collaboration across different social worlds during imperial expansion and its legacy, both challenging and reinforcing geographical spaces and scientific categories. Yet most importantly, such objects were never neutral or equally accessible: they were embedded in extremely asymmetrical colonial power structures that often violently silenced local ecological knowledge and practice. To unpack these dynamics, it is crucial to follow Susan Star's own later work, that urges that “interpretive flexibility without understanding of infrastructure, information needs, standards and classification is a misunderstanding.”^26^ I therefore bring together local historical perspectives with insights from political ecology, focusing on how boundary‐making practices were shaped by specific imperial and ecological entanglements in forms of colonial infrastructure and categorization of life—and how they were sites of cooperation and contestation in multispecies worlds.^27^ In Southern Africa, two interconnected contexts illustrate the relationship between borders and the management of human‐nonhuman relations. First, the regulation and control of movements of livestock, often justified on veterinary grounds. Second, the protection, utilization, and control of wildlife, which gained increasing significance in the context of nature conservation and tourism. Not surprisingly, the two contexts are closely related, as veterinarian fencing often impacts on the movement of wildlife,^28^ while the boundaries of wildlife protection areas are linked to veterinary control.^29^

## 3 Livestock Hierarchies

Veterinarian borders are a crucial feature of colonial expansion and control in Southern Africa, and they determine movements of animals and goods to this day.^30^ Currently, most veterinarian borders are primarily used to control the movement of livestock to prevent the spread of animal disease, and respectively to keep some areas—mostly the formerly white commercial farming areas—disease‐free and allow commercial farmers to access export markets. Today they are mostly marked by fences and control posts along the roads. However, during colonial times—and in the case of Botswana even later—more drastic measurements were taken, such as the instalment of large stock‐free buffer zones, the eradication of certain vector wildlife, the culling of large herds of livestock or the cutting of extensive cut‐lines, where bush was cut and kept open over hundreds of kilometres to prevent some transmitting insects from crossing.^31^ The (veterinary) control of nonhuman animals rests on the understanding that some animals, normally livestock, are valuable, while others, usually predators or vector animals, are considered harmful. Further, hierarchies between different kinds of livestock emerged, often based on their real or assumed danger for transmitting diseases. This process can be illustrated by looking at donkeys crossing the so‐called “Red Line,” a significant colonial veterinary and settlement border in Namibia, which also overlaps with the famous Etosha National Park. ^32^

Donkeys are surprisingly overlooked in Southern African rural history despite their significant role in ecology, agriculture, and economy and their unique feeding patterns, partial resistance to livestock diseases, and affordability.^33^ Within the complex grazing systems in semi‐arid regions, donkeys exhibit unselective feeding habits, consuming even low‐quality forage.^34^ Unlike ruminants, donkeys can quickly ingest large amounts of nutrients, though the food quality may be lower. Moreover, they are more economical and disease‐resistant compared to cattle or sheep, making them essential during crises like droughts and epizootics.^35^ In Namibia's history donkeys held a distinctive role, particularly concerning movements and control of people, goods and animals. Since the late nineteenth century, the Etosha area was strategically significant for colonial powers, serving as a militarized border between the later directly controlled central Namibia (often called “Police Zone”) and the indirectly ruled Owamboland (that became the Ovamboland Homeland under South African rule).^36^ Formal wildlife conservation has deep roots in the area, starting under German rule in 1907 and culminating in the establishment of today's Etosha National Park in the 1950s/1960.^37^

Before the establishment of the park, all traffic between central Namibia and Owambo passed through what is now Etosha National Park. Donkeys played a significant, albeit contested, role in this movement. Throughout most of the twentieth century, donkeys were the only animals permitted to cross the veterinary border (Red Line) between the Police Zone—predominantly private commercial farmland under direct colonial administration—and the northern, indirectly ruled communal lands, which in the colonial economy primarily functioned as an area to recruit migrant laborers.^38^ This specific legal position of the donkey, combined with its relatively low price after the introduction of tractors on commercial farms in the Police Zone, tied it closely to the migrant worker system and defined the donkey's position within the rural economy and society of northern Namibia until today. Migrant workers often purchased donkeys in the Police Zone to transport goods they had bought back home to their families in the reserves or later homelands. Until at least the 1960s, the practice of migrant laborers bringing donkeys from colonial centres to the North led many to perceive these animals as symbols of (colonial) modernity and progress—similar to other consumer goods such as radios.^39^ However, with the introduction of buses to transport migrant labourers and their luggage in the 1950s, donkeys lost their importance as the only animal that was allowed to cross the veterinary border. Nevertheless, imports of donkeys from the Police Zone over the border into the homeland can still be found in the 1970s and 1980s.^40^ One of the reasons for the ongoing interest in donkeys might be the symbolic value of donkeys as being modern; other reasons were that with the growing importance of cars and tractors on commercial farms, donkeys became even cheaper to buy. When, after Namibia's independence in 1990, cars and tractors became more common also north of the Red Line, donkeys gradually lost their status as a symbol of prestige associated with migrant workers and became increasingly linked to poverty.

Still, the new Namibian government estimated the total number of donkeys in Namibia in 1994 at 106,336, with the highest proportion in the central north Owambo regions; by the end of the 1990s this was estimated to be 83,000 animals in this region alone.^41^ Restrictions over and the control of animals from areas north of the Red Line continued in independent Namibia. Whilst the movement of cloven‐hoofed animals southwards remains prohibited, donkeys and horses are still excluded from this rule. Although the Animal Health Act of 2011 reintroduced the need for permits for the movement of all animals, it seems that for donkeys this permit was easy to get; the reason for the permit being stated as for tracing purposes, rather than veterinary ones.^42^

While there is a lot of mobility of people and animals between areas south of the fence and the northern areas of Namibia, the times when migrants brought back donkeys have long gone. However, many of the long historical trajectories of donkeys in the region are still reflected in the present. Despite their role as outsiders, their contemporary association with poverty, and their vulnerability within expanding mixed zones of livestock and wildlife, donkeys have become an integral part of Northern Namibia's rural society. Because of the donkey's particular history, closely linked to veterinary and homeland borders and to migrant workers, it still carries memories of northern Namibia's past. And donkeys will continue to be in a limbo between the *modern* animal that can cross colonial boundaries, and the now *useless* animal that lost its importance to cars, tractors, and machines. In short, the human‐donkey relationship that has developed in the specific context of forceful segregation of people, livestock, and wildlife can also be regarded as an important contribution to the ongoing debates on companion species—and critiques of it—as well as the extensive academic engagement with animal labour under capitalism.^43^ Or in other words, the donkey's shifting position—from an animal associated with mobility and modernity to one increasingly linked to poverty—shows how regimes of valuation, often applied to charismatic versus non‐charismatic wildlife, also hierarchize livestock, rendering the same species alternately desirable or expendable across time and space.

The case of the veterinarian and intra‐colonial border of the Red Line, yet again, shows the impact of borders on the role and position of a specific species in the rural societies of northern Namibia. However, it was not only veterinary reasons—and borders—that impacted on the hierarchies of livestock in colonial and apartheid Namibia. In the very South of the country, the Karakul sheep gained some interest in Namibian historiography over the last years.^44^ Again, like the donkeys, the history of Karakul is tightly linked to borders and labour.^45^ Karakul, originating from Central Asia, were introduced to Namibia during German colonial times, and with rising prices for Karakul pelts on the international markets, the animal became a commercially highly successful good in the arid areas of Southern Namibia from the 1920s until the 1970s. Without going too deeply into the complex relationships between agricultural labour, technology, and the rivalries involving South Africa, its mandated and later occupied territory of Namibia, and other colonial neighbours, the following section highlights the impact of colonial borders on Karakul sheep.^46^

In 1929, at the height of Karakul breeding in Southern Namibia, the administration in Windhoek banned the export of any Karakul sheep, even to South Africa.^47^ This ban was only lifted for the export to South Africa for state exports in 1945 and for private exports in 1957.^48^ The ban was motivated by the need to protect the highly lucrative Karakul pelt market, which was seen as one of the few viable options for the so‐called “poor white” farmers settled on the newly subjugated and ecologically challenging lands of Southern Namibia. Additionally, the government had made significant investments in the knowledge and infrastructure required for the Karakul industry.^49^ As Bernard C. Moore shows, the Karakul production in Namibia also led to a demand for labour, initially sourced locally and later from Portuguese Angola. The ability of the local workers to leave abusive employers and return to local reserves encouraged white farmers to hire Angolan migrants, viewing them as the future workforce for shepherding. Between 1926 and 1970, at least 225,000 labour contracts were signed by Angolans entering Namibia.^50^

The Karakul industry in Southern Namibia is therefore interesting in regard to bordering processes. First, while the movement of Karakul over the borders was strictly prohibited—in the case of Angola even until the 1970s—the people who looked after Karakul often came from outside of the territory. Secondly, for the first few decades after South Africa's take‐over of Namibia, the ban of Karakul exports was one of the reasons for upholding a border between South Africa and the commercial farming areas of Namibia. For most other animals, as well as for whites of both territories, this border became irrelevant, because South Africa increasingly aimed at turning the Namibian Police Zone into an integral part of its territories.^51^

The valuation of Karakul and, to a certain degree, the people looking after them has changed over time. From the perspective of the settlers, Karakul was of such high value that it was worth closing the border for the export of the rams and importing migrant laborers from as far as Angola. With the decline in pelt prices in the 1970s, Karakul lost its economic viability, and there was no longer a need for it to be exclusively maintained for the white Namibian farmers. Eleanor Schaumann has shown how Karakul later also became owned by Africans, and despite no longer being of much financial value, the sheep kept a high emotional value even after Namibia's independence. In 2012, the term “Swakara” (South West African Karakul), which had been used to market Namibian Karakul pelts since 1966, was officially adopted for the Namibian Karakul breed. This change aimed to differentiate it from Central Asian Karakul farming practices. Genetic testing supported this decision, revealing that Swakara sheep were genetically closer to Namibian breeds like the Namaqua sheep than to other Karakul sheep, for example, those from Germany.^52^ So finally, more than a century after being imported to Namibia, it was no longer physical borders that tied Karakul to Namibia, but its redefinition as a national Namibian breed. In this way, the political borders that once kept Karakul in Namibia have re‐emerged as biological boundaries—as the breed is now genetically defined as Namibian, even after its economic relevance has faded.

## 4 Wanted Wildlife and Unwanted Weed

(Post‐)colonial borders and border‐making not only affect livestock but also play a significant role in wildlife and plant management. The border between Namibia and Botswana along the Chobe River illustrates how shifting border regimes, together with changing economic and security policies, have produced inter‐ and intraspecies hierarchies, resulting in violent conflicts over the past decades.

The border along the Chobe River is both a contested national boundary and a separator of the strictly controlled Chobe National Park in Botswana from communal lands in Namibia. The border that lies within a large and often‐changing riverbed has given rise to territorial disputes, particularly around the small river island Sedudu/Kasikili, as well as to an ongoing, often violent conflict involving the movement of both animals and people across the border.^53^ The Chobe's fluctuating water levels make crossings possible for most animals and for people—at least during periods of low water. The small but fertile Sedudu/Kasikili Island was historically used for cultivation and habitation and later gained particular economic value when it became integrated into the Chobe National Park. Initially marking a frontier between amicable imperial administrations, the Chobe border became, during Namibia's and Zimbabwe's liberation wars, a site of violent struggles and a boundary between independent Botswana and apartheid South Africa's occupied Namibia. Since Namibia's independence the border increasingly became a site of “green violence.”^54^

The Chobe River clearly illustrates how nature has historically shaped and been shaped by border‐making through practices of inclusion and exclusion that affect certain people and animals and continue to produce violence today.^55^ On the Namibian side of the border, communal land supports fishing, farming, and livestock herding, while most local farmers so far have not benefited from wildlife‐tourism.^56^ On the Botswana side, the government has been pushing for wildlife conservation and luxurious tourism since the 1960s, and has therefore been protecting its main assets—the so‐called charismatic mega‐fauna (e.g., elephants, buffalo, and large cats). To protect the border from (alleged) poachers, the Botswana Defence Force (BDF) has heavily patrolled it, reportedly killing over fifty suspects since Namibia's independence in 1990.^57^ Shortly after its formation in 1977, the BDF began to integrate wildlife conservation into its training, recognizing conservation areas as national security threats and intertwining conservation with border security.^58^ Across the border, small‐scale farmers face constant threats of harassment or even shootings by the BDF, while also dealing with increasing incursions of large wildlife and predators into their villages and fields.

To understand how this border has shaped the hierarchization and valuation of species, it is worth examining animal crossings and the responses to them by local people and administrations on both sides over time. In 1890, the Anglo‐German Treaty defined the river as the divider of the sphere of influence of the British and the German colonial empires.^59^ However, only at the very end of the German colonial period, in 1912, was the area mapped for the first time and the border through the Chobe swamplands formally demarcated.^60^ Until at least that point in time—and in many respects even beyond—the river did not function as a significant dividing line in the lives of human and nonhuman inhabitants. Most people living on both sides identified as Basubia, a group of people who were for a long time under the control of—or at least in close connections with—the Bartotse Kingdom in today's Zambia. The region was sparsely settled, with people, livestock, and wildlife mostly moving freely across the river. Particularly, inhabitants from the northern shore also occasionally inhabited and regularly planted on the small islands within the river.^61^

While wildlife, livestock and people might have moved over the river freely, this never meant that there were no conflicts around grazing, planting, fishing, and hunting, as well as about the political belonging of the people living in the area. These conflicts were, as an old local farmer remembered, “the normal talks and scuffles in a rural community, not the causes of the presidents’ fighting.”^62^ The dynamic of such conflicts shifted with the increasing influence of colonial power along the river, coupled with ongoing discussions over the precise demarcation of the border. Tensions heightened following the establishment of the Chobe National Park and Botswana's independence in the 1960s. When the border still separated two farming areas under largely cooperative colonial administrations, its exact course was primarily a matter of administrative concern. For local people and animals, it was only relevant in the context of routine conflicts over livestock grazing across the river or white trophy hunters illegally crossing in pursuit of game.^63^

This all changed in 1960 when much of Botswana's riverfront, and with it Kasikili/Sedudu, the most contested island in the river, became part of Chobe Game Reserve, later renamed Chobe National Park; and only six years later, in 1966, the border no longer separated colonial governments but independent Botswana and apartheid‐era South Africa. The creation of Chobe Game Reserve transformed the river into a conservation border, dividing strict conservation and tourism from communal farming. Within this changing legal and economic context along the border, people and animals adjusted their cross‐border movements; protected animals, such as elephants or lions, crossed the border and became a threat to farmers on the Namibian side of the river. Rather than simply ignoring shifting economic, political, and spatial conditions along borders—or being passively shaped by them—both humans and nonhumans have historically responded to, interacted with, and co‐constituted such evolving border regimes. People living in the Caprivi continued to enter the park to hunt and fish—now illegally—or to bring back cattle that ran away. However, tensions soon extended beyond disputed islands and animal movements. With the beginning of the war of independence in Namibia in 1966, conflicts stirred around people and weapons crossing an increasingly militarized border into what soon became a war zone.^64^

However, even during the wars of liberation in Namibia and Zimbabwe, which also sometimes spilled over to northern Botswana, nonhuman animals continued to cause conflict—and sometimes also collaboration—along the river. During the Namibian War of Liberation, the occupying South African Defence Force (SADF) invested in nature conservation in the area, both for economic and for strategic reasons, at the same time its members were massively involved in large‐scale destruction of wildlife.^65^ Meanwhile, newly independent Botswana kept its prime Chobe National Park open and in 1977 established the BDF whose stated goals were not only to secure the border against unwanted soldiers and independence fighters from the neighbouring countries, but also to protect wildlife in and around Chobe.

Interestingly, it was not only soldiers, hunters, livestock, and wildlife crossing over the rivers that caused concern, but also *Salvinia molesta*, an invasive water‐weed that overgrew the Zambezi and Chobe rivers after the building of the Kariba dam in 1959 farther down the river.^66^ The plant prompted both sides to act, as officials on the Namibian side feared major threats to the fishing economy, while on the Botswana side the administration was concerned with loss of the area's attractiveness to tourists. Military officers on both sides feared that the weed's rapid spread could ease insurgent border crossings, because the rapid spread of the weed along the riverbanks made the border increasingly difficult to patrol and provided cover that facilitated hidden river crossings. In response, despite Botswana's opposition towards South Africa's white apartheid rule and its occupation of Namibia, military personnel, ecologists, and administrative officers of both countries collaborated to eliminate the plant. The South African air force provided planes to track its spread, closely working with counterparts in Botswana.^67^ Bollig and Vehrs showed that this cooperation in the midst of severe diplomatic rifts not only worked for the eradication of unwanted weed, but similarly for the eradication of another unwanted species, the tsetse fly.^68^ Both countries used their resources, technologies, and personnel to create a border that supports a nature that is clearly divided into undesirable elements, such as poachers, invasive species like the water weed or tsetse flies, and desirable charismatic wildlife and tourists. In this sense, nonhuman actors did not merely cross or challenge an existing border but actively participated in its reconfiguration: invasive plants, wildlife movements, and disease vectors triggered new forms of cooperation, surveillance, and exclusion, thereby materially and politically remaking the border itself. The historical hierarchization of animals also shapes the border to this day: some animals (and groups of humans) are allowed to push the borders, to cross them, and even to create new spaces in which to thrive—or, to borrow Rees’ words again, to “contribute to their futures”—while others are still denied any agency, and their movements and interactions with and across the borders are heavily, often violently, restricted.^69^

Since Namibia's independence, the two countries have officially resumed their status as friendly neighbours. This was true for the general relationship of the two countries but did not apply to the Chobe border. While the dispute over Kasikili/Sedudu Island escalated and both countries reinforced their military presence, the end of the war and apartheid raised expectations of increased tourism—prompting further protection of Chobe National Park's key attraction: its charismatic megafauna. In 1999, the International Court of Justice ruled that Kasikili/Sedudu belongs to Botswana, and, as had already been declared in 1966, it was immediately incorporated into Chobe National Park.

Nevertheless, the region still had a high presence of military and security forces, with a difficult‐to‐control national and conservation border in an ever‐changing, swampy riverbed. Under the presidency of Ian Khama (2008–2018), Botswana intensified conservation enforcement, promoting high‐end tourism and a shoot‐to‐kill policy against alleged poachers. On the Namibian side, residents continue to report killings, harassment and increasing incursions of elephants and buffaloes onto their fields. Many fear gradual displacement to create a de facto buffer zone for Chobe National Park—an idea floated since the 1970s.^70^ While there is no official confirmation of such plans, people's experiences are stark, and many state they are treated “worse than animals.”^71^

Conflicts have also increased with the growing number of elephants, buffaloes, and some large predators crossing over the increasingly unprotected rivers into Namibia. A fisherman and game guard in Kabulabula explained that the entire system of interaction between and within humans and animals and even plants has changed, or in other words, that the region is experiencing a significant shift in inter‐ and intra‐species interactions. An increase in Big Five wildlife crossing the border is leading to the destruction of fields and large trees, threatening livestock and biodiversity. The fisherman described how the rising number of elephants has transformed the landscape beyond recognition; large trees are destroyed and fields and grazing areas, which they have to give up, are encroached by smaller bushes. At the same time, fishing in the river has decreased due to the attacks by BDF, leading to higher numbers of fish and crocodiles in the river. An elderly person in Kabulabula concluded: “I am missing the large herds of small, less noticeable species that once lived with us, that fed from our unused fields. Today we only have elephants, buffaloes, wildebeests and lions dominating our land and our nature.”^72^ While not all of these observations may hold ecologically, and not all “new” animals cross the border, it's striking that many in the area experience the overconcentration of certain species as a loss for others—not just livestock, but also less “charismatic” and economically less profitable species like small antelopes and trees.

Finally, changes in nonhuman hierarchies also reflect shifts in human social hierarchies. These days, many people are moving away and giving up farming, leading to a concentration of land and wealth in the hands of a few. Larger farmers, who can afford herders and better protection, are taking over the land. Many others are left with little choice but to move away or take unskilled jobs in the mushrooming lodges that aim to attract ever more spectacular wildlife. Today the Chobe River is a border that is open to wealthy tourists and charismatic wildlife, but increasingly closed to Black people, their livestock, and non‐charismatic animals.

## 5 Conclusion

Examining these border contexts reveals how borders profoundly shaped multispecies hierarchies. Namibia's Red Line, Karakul sheep management, and the conservation and national border along the Chobe show how (post‐)colonial borders not only defined human territories and movements but also imposed regimes privileging some groups or species while marginalising others. Such regimes often reinforce capitalist and colonial logics that value specific animals over others based on both their economic utility and their social value. The colonial legacy shaped ongoing human‐animal interactions at borders, seen in the veterinary Red Line that separates livestock in Black communal areas from livestock on mostly white commercial farms, and along the Chobe River, where conservation often prioritises charismatic megafauna over local communities and their livestock.

Along borders, the hierarchisation of nonhumans always interacts with the hierarchisation of human groups—be they migrant labourers, herders or alleged poachers—and resonates with logics that were central to apartheid and colonialism. Including nonhumans in society without addressing (local) power structures risks undermining the hard‐won humanity of formerly oppressed groups. Neo‐colonial conservation logics, rooted in violence and exclusion, reinforce hierarchies where certain animals—and by extension, certain humans—are valued, while others are deemed expendable. Ultimately, this study calls for critically reassessing the boundaries we draw, both geographically and species‐wise, and to challenge the underlying logics that sustain them.

The colonial and apartheid state's demarcation of *wild* and *domesticated*, of *vermin* and *game*, and of human populations across ethnic and class lines, also operated as interlinked bordering practices and were mutually reinforcing. Borders, fences, and veterinary cordons functioned as material expressions of these divisions, shaping who could move, stay or survive under what conditions, while at the same time it was human and nonhuman movements that shaped and contested such borders and boundaries. Therefore, an engagement with local histories cautions against a conceptual weakening of human‐nonhuman boundaries and the relative capacities of different species, to take up once more Amanda Rees’ poignant phrase, to shape the future, especially in colonial contexts where species hierarchies placed some animals above many people, with dehumanisation central to racialised scientific discourses.

The analysis presented here underscores the importance of approaching multispecies entanglements along borders not as abstract conditions, but as historically and politically situated, and within specific academic debates. Focusing on Southern Africa's borderlands showed how interspecies relations were forged in unequal geographies of power, where colonial and apartheid infrastructures calibrated life and death along interlocking lines of species, race, and territory. As I highlighted earlier—referring to Star's own engagement with her concept of boundary objects—it is also the attention to the infrastructures, classifications, and standards that shape practice—particularly where colonial regimes of ordering life continue to exert power over humans and nonhumans. Thus, taking nonhuman mobilities seriously means tracing their effects on borders, but also recognising how borders themselves help naturalise deeply political—often racist—distinctions between species. Veterinary fences and conservation boundaries are therefore not just environmental infrastructures; they are instruments of multispecies ordering whose enduring legacies for both human and nonhuman life demand critical engagement with the material and epistemic foundations that sustain them.

## Conflict of Interest

The author declares that there is no conflict of interest.

## Footnotes

1 On commodification, see Harvey 2003; Castree 2003. On new biological conceptualizations of animals, see, e.g., Thomas 2014; on plants: Calvo et al. 2020.

2
Thomas 2014; Moore 2017; Ritvo 2007; Weitering 2020; Gillespie and Collard 2015; O’Gorman and Gaynor 2020.

3
White 1991 and White 2000.

4
Swyngedouw 2014; Hersey 2019.

5
Hersey 2019 on 6–9; see also Yusoff 2018.

6 See for some of the most influential contributors to these debates: Said 1993; 74; 60; 52.

7 See, e.g., Tsing 2015; Roy 2015.

8 See also: 39; 93. This also includes a more localized analysis of the drivers and complex layers of imperialism and colonialism in the context of Namibia and Botswana. For a discussion in the case of Namibia, see 36 and 46.

9
Beinart and McGregor 2003.

10
Mbembe 2015 shows how equating Blackness with the nonhuman served as a foundational logic throughout Europe's imperial expansion for justifying racial oppression and exclusion.

11
Fanon 1963 (translation of the French original of 1961), on 7.

12 On race and conservation in Southern Africa in general: Musavengane and Leonard 2019; Koot et al. 2024. From a historical perspective: Beinart 2008; Dlamini 2020. For East Africa under German colonial rule: Gißibl 2022.

13
Mavhunga 2011; Cloete 2011.

14
Mavhunga 2011; van Sittert 2016; Adhikari 2011. For a convincing historical overview of colonial and post‐colonial conservation practices and their role in territorializing the continent from an Ethiopian perspective, see also Blanc 2020.

15
Rees 2017, on 9.

16
Deckha 2012, on 530.

17
Dube 2019; Olwig et al. 2019; Fleischmann, preprint. See for a discussion of African borders and borderland studies: Coplan 2010; Ramutsindela 2019. On Covid‐19: Sivasundaram 2020; Radil et al. 2021.

18
Pratt 1992; Haraway 2008. See also: Kirksey and Helmreich 2010; Wilson 2019; Isaacs and Otruba 2019.

19
Fleischmann, preprint; Mavhunga 2011; Guerrini 2016.

20
Ozguc and Burridge 2023.

21 See, e.g., Gillespie and Collard 2015; Thomas 2014; Kirksey and Helmreich 2010; for a critical overview, see, e.g., Chipato and Chandler 2024.

22
Abrell 2021; Evans 2021; Barla and Hubatschke 2020.

23
Gutkowski 2021, on 135.

24 See Gutkoswki 2021; Brito 2024.

25
Star and Griesemer 1989.

26 Susan L. Star, “The History and Boundaries of Boundary Objects,” conference at the workshop “Boundary Object, Boundary work,” Grenoble University, 13 September 2007, n.p. See also Trompette and Vinck 2009.

27 For a similar approach, see also 31.

28
Mbaiwa and Mbaiwa 2006; Gadd 2012.

29
Ferguson and Hanks 2012.

30 There is a large body of literature on veterinarian history in Southern Africa that often includes veterinarian fencing. For an overview, see, e.g., Mwatwara 2024.

31 For an overview of the different methods to keep wildlife and livestock separated, see van Dam et al. 2024.

32 See also Lenggenhager et al. 2024.

33
Jacobs 2001.

34
Samuels et al. 2016.

35
Jacobs 2001.

36
Miescher 2012.

37
Dieckmann 2007; Miescher 2012.

38 See, e.g., National Archives of Namibia (hereafter NAN), Agriculture and Veterinary Services Branch, 90‐V1/1 v2, Senior Veterinary Officer to Magistrate Outjo, 11 July 1924.

39
Lenggenhager et al. 2024.

40 E.g., NAN, Ovambo Government Agriculture (hereafter OVA), 6‐20‐2, Director of Agriculture to Bantu Commission, 1 March 1974 or NAN, OVA, 6‐9‐1, Report by C. F. Watson, Director Agriculture and Forestry, to the Native Administration (Department of Cooperation and Development), 19 February 1981.

41
National Planning Commission 1997; National Planning Commission 2004.

42
Namibia Animal Health Regulations (2011): Animal Health Act, Pub. L. No. 1, 358, 53, online: https://archive.gazettes.africa/archive/na/2011/na‐government‐gazette‐dated‐2011‐04‐20‐no‐4694.pdf (accessed 24 July 2026).

43
Haraway 2008. For a critique of Haraway's concept, see 44. On the extensive academic engagement with animal labour in capitalism, see 38; 82; 19; in African history more generally: 84.

44
Moore 2021; Swanepoel 2023; Schaumann 2024; Moore and Lenggenhager 2025. For a perspective from Angola, see also Saraiva 2014.

45
Swanepoel 2023; Moore 2021.

46 For the (agricultural) histories of Southern Namibia, see, e.g., Moore 2025.

47 A less strict ban was already introduced in 1925 (Swanepoel 2023, on 106) and later extended to all sheep with some kind of Karakul ancestry (Moore 2021).

48
Moore 2021.

49
Swanepoel 2023.

50
Moore 2021, on 95.

51 See also Rutishauser 2023.

52
Schaumann 2024, on 2.

53 For an overview of the history of the border, see also Lenggenhager and Mogende 2025.

54
Büscher and Ramutsindela 2016, on 2.

55 See also Ramutsindela 2015.

56
Kalvelage et al. 2023.

57
Mongudhi et al. 2016.

58
Death 2016.

59 Anglo‐German Treaty (Heligoland‐Zanzibar Treaty), 1 July 1890, online: https://jusmundi.com/en/document/treaty/en‐agreement‐between‐great‐britain‐and‐germany‐respecting‐zanzibar‐heligoland‐and‐the‐spheres‐of‐influence‐of‐the‐two‐countries‐in‐africa‐1890‐anglo‐german‐agreement‐of‐1890‐tuesday‐1st‐july‐1890 (accessed 24 July 2026).

60 Botswana National Archives and Records Services (BNARS), Secretariat (S.), 35/12: Report on the main channel of the Linyanti (or Chobe) River, 5 August 1912.

61 On the region's nineteenth‐ and early twentieth‐century history, see 27; 3.

62 Interview done by the author, Impalila Island, 8 December 2022. All interviews for this section were conducted by the author in connection with a broader research project (2022–2024) and all interviewees have been anonymized due to the sensitivity of the topic. The use of all material follows established research ethics and journalistic codes of conduct in the relevant countries.

63
Lenggenhager and Mogende 2025.

64 See also Kangumu 2011.

65
Lenggenhager 2018; Bollig et al. 2024.

66
Bollig and Vehrs 2021; Lenggenhager 2018, on 112–116.

67
Bollig and Vehrs 2021.

68 Ibid.

69
Rees 2017, on 9.

70 Map by Ben C. W. van der Waal, 1976, NAN, Caprivi Agriculture and Forestry (CAF), 6/19.

71 Interview by the author, Kabulabula, 10 December 2022.

72 Ibid.
